# Breast and cervical cancer screening services in Malawi: a systematic review

**DOI:** 10.1186/s12885-020-07610-w

**Published:** 2020-11-12

**Authors:** Chiara Pittalis, Emily Panteli, Erik Schouten, Irene Magongwa, Jakub Gajewski

**Affiliations:** 1 Department of Public Health and Epidemiology, Royal College of Surgeons in Ireland, Beaux Lane House, Lower Mercer Street, Dublin 2, Ireland; 2 Management Sciences for Health, P/BAG 398, Lilongwe, Malawi; 3 Institute of Global Surgery, Royal College of Surgeons in Ireland, Beaux Lane House, Lower Mercer Street, Dublin 2, Ireland

**Keywords:** Breast Cancer, Cervical Cancer, Cancer screening, Early detection of Cancer, Public health, Malawi

## Abstract

**Background:**

To identify and to assess factors enhancing or hindering the delivery of breast and cervical cancer screening services in Malawi with regard to accessibility, uptake, acceptability and effectiveness.

**Methods:**

Systematic review of published scientific evidence. A search of six bibliographic databases and grey literature was executed to identify relevant studies conducted in Malawi in the English language, with no time or study design restrictions. Data extraction was conducted in Excel and evidence synthesis followed a thematic analysis approach to identify and compare emerging themes.

**Results:**

One hundred and one unique records were retrieved and 6 studies were selected for final inclusion in the review. Multiple factors affect breast and cervical cancer service delivery in Malawi, operating at three interlinked levels. At the patient level, lack of knowledge and awareness of the disease, location, poor screening environment and perceived quality of care may act as deterrent to participation in screening; at the health facility level, services are affected by the availability of resources and delivery modalities; and at the healthcare system level, inadequate funding and staffing (distribution, supervision, retention), and lack of appropriate monitoring and guidelines may have a negative impact on services. Convenience of screening, in terms of accessibility (location, opening times) and integration with other health services (e.g. reproductive or HIV services), was found to have a positive effect on service uptake. Building awareness of cancer and related services, and offering quality screening (dedicated room, privacy, staff professionalism etc.) are significant determinants of patient satisfaction.

**Conclusions:**

Capitalising on these lessons is essential to strengthen breast and cervical cancer service delivery in Malawi, to increase early detection and to improve survival of women affected by the disease.

## Background

Cancer incidence is increasing in Malawi [1]. Breast and cervical cancer are among the most common cancers affecting Malawian women, of which cervical cancer is the most frequent cancer diagnosis, with daunting numbers of new cases every year (4163 new cases just in 2018 [2]). As an AIDS-defining condition, risk factors for cervical cancer are considerable due to the high HIV prevalence in the country, 10.6% of adults aged 15–64 years [3, 4]. Breast cancer is the third most common female cancer, with 1216 new cases diagnosed in 2018 [2].

Breast and cervical cancer outcomes and survival tend to be poor due to a combination of late presentation of symptoms to a healthcare facility, late stage at diagnosis, and limited access to timely and standard treatment [5]. A staggering 80% of cervical cancer hospital admissions in Malawi present at inoperable stages [6]. Both breast and cervical cancer are associated with high morbidity and mortality in Malawi; the median survival time from the time of diagnosis is 9.6 months for cervical cancer and 5.6 months for breast cancer [5]. Hence, early detection services are urgently needed in Malawi in order to facilitate cancer downstaging and timely treatment, and improving chances of survival for patients.

The Malawi Ministry of Health called for screening to be integrated in primary health care and routinely offered to all women [6, 7]. However, a recent assessment found that these services are not widely available and have very limited capacity [6].

A national Cervical Cancer Control Programme (CECAP) has been in place in Malawi for over a decade, focusing on a screen-and-treat approach employing inspection with acetic acid (VIA) and cryotherapy for lesions, but implementation has been challenging^6^ (in 2015 coverage was 27.3%, well below the target rate of 80% [8]). In regards to breast cancer control, despite its inclusion among the priorities of the National Sexual and Reproductive Health and Rights Policy (SRHR) 2017–2022 [9], a national programme has not materialised. Some initiatives have emerged (*see the studies in our review*) but in a sporadic manner, with no coordination among them or links with national actors. Hence the importance of assessing progress made and documenting lessons learned to-date. Cancer patients in Malawi are young, with a mean age of 33 years [4], meaning effective screening programmes could and would save years of life.

In Malawi poverty and inequality remain stubbornly high across the country. The majority of its 17.5 m population (84%) resides in rural areas [10], where it is estimated that one in two people are considered poor [11]. The local economy is largely reliant on subsistence agriculture and the informal sector, of which women are a major contributor. However, whilst women make up over half of the population [10], serious gender disparities still exist in terms of access to health care [12], despite a significant burden of disease: women account for 55.9% of all new registered cancer types [1]. Improving access to essential cancer services is crucial for local development and to protect those most vulnerable to poverty and disease.

Government facilities (primarily the Malawi Ministry of Health and Population) cater for the health of the majority of citizens, however the private for profit and private not for profit (comprised of mainly religious institutions and non-governmental organisations) sectors are growing contributers [13].

The principal objective of this systematic review was to comprehensively assess breast and cervical cancer early detection services in Malawi by answering the following research questions:

1. What is the state of the delivery of breast and cervical cancer screening and early diagnosis services in Malawi in terms of accessibility, uptake, acceptability and effectiveness?
2. What factors either propel or hinder breast and cervical cancer service delivery in Malawi?

## Methods

We conducted a systematic review of published scientific literature on breast and cervical cancer early detection services in Malawi following the methodological approach described by Petticrew and Roberts [14], and the Preferred Reporting Items for Systematic Reviews and Meta-Analyses (PRISMA) approach [15] as the reporting framework.

### Selection criteria

We followed the Population, Concept and Context (PCC) framework [16] suggested by the Joanna Briggs Institute as a tool to guide literature reviews. Inclusion/exclusion criteria are summarised in Table 1

**Table 1:** Population, concept and context (PCC) framework

	Inclusion Criteria	Exclusion Criteria
Population	Patients and staff involved in breast and/or cervical cancer screening services	Screening for other cancers Breast/cervical cancer treatment services only
Concept	Uptake, feasibility, acceptability and effectiveness of breast and/or cervical cancer screening services	Papers where breast and/or cervical cancer screening is mentioned but the main focus is not on assessing service delivery or not on screening (e.g. papers about breast/cervical cancer prevalence rates only or epidemiology, and factors affecting patients’ health seeking behaviour in general with no reference to services)
Context	Service delivery at health facilities	Service delivery in the community

The search focused on any studies related to the provision of breast and/or cervical screening services in Malawi, available in the English language. There was no publication date cut-off or study type limitation (only text and opinion papers were omitted). This inclusive approach allowed for examination of the totality of available literature.

#### Search strategy

Search strings were created in collaboration with a bibliographic search expert using the Peer Review of Electronic Search Strategies 2015 Guideline Evidence-Based Checklist (PRESS) [17]. To maximise the reach of our search we used combinations of medical subject headings (MeSH) related to early detection of cancer of the breast or cervix and key words related to cancer (e.g. tumour, neoplasm, cancer), the organ of interest (i.e. cervix, uterine, breast) and detection (e.g. early diagnosis, screening, detection etc.). The search string was originally developed and tested in June 2019 in one database and then adapted for the others. Detailed search strings used are reported in Appendix. The search strategy was executed across the following bibliographic databases: Embase, PubMed, Ovid MEDLINE, The Cochrane Library, Global Health and African Journals OnLine (AJOL). This was complemented by thorough grey literature retrieval mechanisms, including searches in Google search engine, using a similar keyword search strategy, and manual revision of reference lists of retrieved articles with the aim of identifying additional relevant articles.

#### Document management and screening

Search results were merged in EndNote version X7 to facilitate management, as well as identification and removal of duplicates. They were then uploaded into Covidence, an online platform for systematic literature reviews which supports researchers in screening, quality appraisal and analysis of retrieved papers, while maintaining accurate records of decisions made.

The screening process was performed using the pre-specified selection criteria as reported in the PCC Table 1. Two researchers working independently reviewed each title and abstract, with a third researcher acting as mediator in the case of conflicting opinions. The full text of the studies identified as potentially relevant was retrieved and the same approach was followed for their review, with two researchers assessing the studies and any disagreements settled with the help of a third reviewer. If a paper was excluded, reasons for exclusion were recorded as follows: i) full text unavailable; ii) text/opinion/poster; iii) wrong focus of study. Each study was then assessed for methodological rigor and risk of bias using The Joanna Briggs Institute Critical Appraisal tools [16].

#### Data extraction

Data extraction from the final selection of studies included in the review was done manually. Bespoke tables were developed by the researchers in MS Excel, keeping in mind the focus and objectives of the review. We mapped any factors mentioned in the included papers as having an effect on cancer services, including: barriers to screening, barriers to referral/treatment, cancer screening uptake (coverage), existence of awareness raising intervention, patient experience of screening, and initiatives to bundle cancer screening programmes with other services. We then organised these factors affecting the delivery of breast and cervical cancer early detection services into patient-level, health facility-level and healthcare system-level factors as described in the following section.

## Results

**Fig. 1:**
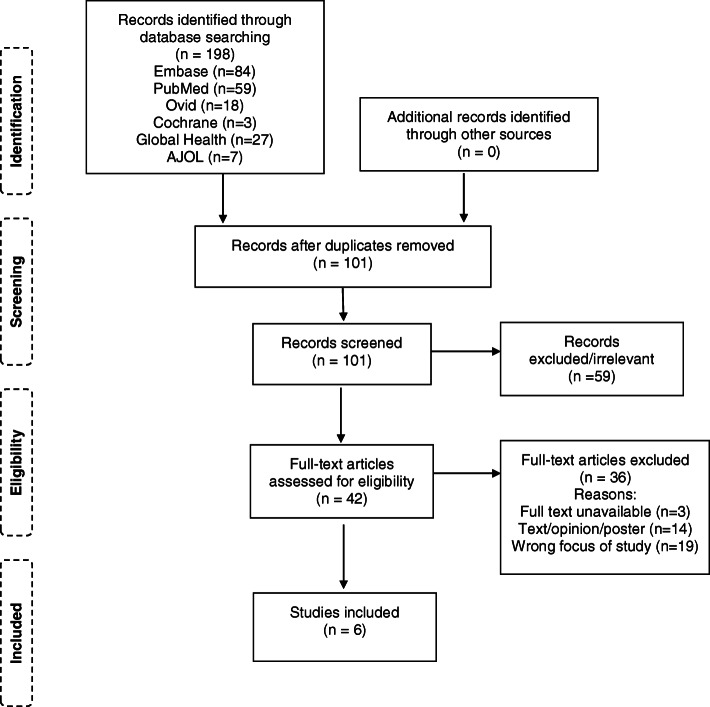
PRISMA flow diagram

The bibliographic database search retrieved 198 papers, with no additional studies identified from grey literature. After removing duplicates, 101 studies were screened based on title and abstract. Of these, 42 were deemed eligible for a full text assessment. The final selection of studies included in this review consists of 6 articles. A flow diagram of the screening process is illustrated in Fig. 1.

Publication dates of included studies ranged between 2015 and 2018. Only two papers [18, 19] were identified in relation to breast cancer early detection services in Malawi. They assessed different aspects of a breast cancer screening pilot programme conducted in Lilongwe, involving the use of trained laywomen to educate urban women about breast cancer and conduct clinical breast examination (CBE) screening, integrating it with other health services in diverse clinical settings.

Four of the included studies were about cervical cancer screening services delivered under the national cervical cancer programme supported by the Ministry of Health. Of these, three [8, 20, 21] examined the national cancer control programme, with Maseko et al. 2015 [20] and Msyamboza et al .[8] focusing on implementation challenges and Maseko et al. 2014 [21] investigating client satisfaction with the services. The fourth and final study was conducted by Pfaff et al. [22], and reported on the experience of integrating cervical cancer screening and treatment into HIV services at Zomba Central Hospital. While this integration of services is recommended by the national programme, it is still not fully operational, therefore the paper reported on process and outcomes of integration of cervical cancer screening with HIV care at the hospital, offering lessons for other facilities wishing to do so.

All of the study designs were observational and descriptive in nature. In general, the quality of the evidence according to the international GRADE guidelines was low [23]. A summary of the final selection of studies included in the review, as well as details on study rigour and risks of bias are reported in Table 2.

**Table 2:** Summary of included studies

Author / Year	Study participants	Study setting	JBI level of evidence	Study description	Study rigour
Gutnik et al. 2016 [18]	1220 women age ≥ 30	5 urban health clinics in Lilongwe	Level 3.e	Pilot study evaluating feasibility and acceptability of CBE screening performed by laywomen. 4 months (January to April 2015) quantitative descriptive study.	Laywomen were paid, so success may not be comparable to unpaid volunteers. Urban area, may not be applicable to rural areas. The intervention targeted participants already attending clinics so already demonstrating health-seeking behaviours, may not be applicable to the entire population. Transport reimbursements and telephoning referred women was likely to have contributed to the high rates of completed referrals. No control group. Performance in rural areas, effects on cancer stage and mortality, and cost effectiveness require evaluation.
Kohler et al. 2017 [19]	25 women screened for breast cancer	5 urban health clinics in Lilongwe	Level 3.e	Qualitative study to explore perceptions and experiences of Malawian women who underwent CBE screening performed by laywomen.	Participants were already attending clinics already demonstrating health-seeking behaviours, so may not be applicable to the entire population. Most participants lived in urban/peri-urban areas and were more educated compared to the general population, so may not be applicable to the entire population. No data was collected prior to the educational talk, therefore changes in knowledge could not be formally assessed. Interviews took place up to 5 months after a participant’s screening, so could be subject to recall bias. Interviews were conducted by the same laywomen participating in the intervention delivery, with some risk of response bias
Maseko et al. 2015 [20]	41 service providers and 9 district health coordinators	1 central hospital, 7 health centres and 13 district hospitals	Level 3.e	Mixed methods study exploring health system gaps responsible for the poor performance of cervical cancer screening and treatment services in Malawi	Only 14 out of the 29 administrative health districts in Malawi were in the sample, may not be generalisable. Participants filled the questionnaires under the guidance of trained research assistant, possible response bias
Maseko et al. 2014 [21]	120 women screened for cervical cancer	16 public health facilities	Level 3.e	Descriptive study to examine the experience of women who have been screened for cervical cancer at public health clinics. Data collection Mar-Jun 2013 using a semi-structured questionnaire	Exit interviews were conducted only over 1 day at each facility. Clinics had multiple service providers and it was possible for facility managers to allocate the best health care provider to do the screenings on that day when the researcher conducted the interview. Patient satisfaction surveys prone to response bias, especially in the form of face-to-face interviews and low education level of respondents. Participants were not randomly chosen
Msyamboza et al. 2016 [8]	145,015 women screened for cervical cancer	All clinics participating in the national cervical cancer control programme across Malawi	Level 3.e	Cohort study assessing the national cervical cancer control programme, for the period 2011–2015	Limitations related to use of health facility data, including incompleteness and bias (it includes only information obtained from people who come to health facilities or clinics). High loss to follow up, missing data.
Pfaff et al. 2018 [22]	957 HIV-positive women screened for cervical cancer	1 HIV clinic in Zomba Central Hospital	Level 3.e	Descriptive analysis of an NGO-led intervention. Study period May 2016 to March 2017. The methodology seems to suggest only a quantitative analysis of patient data, but anecdotal evidence about the service reported across the document	Participants recruitment process not clear in the paper. Authors mention that screening services were offered by Expert Clients (ECs), who are patients on ART, but no details on when/how women were approached, if EC were trained, if informed consent was sought etc. Another part of the document suggests that it is the clinicians who refer women to the cancer screening service, while the ECs accompany them to the screening room. No cost effectiveness evaluation of the intervention or information on sustainability. All participants were already demonstrating health seeking behavior as they were already attending the HIV clinic, thus this may not be generalisable to the entire HIV positive population in Malawi. The study reports the number of HIV-positive women attending the clinic who were screened for cervical cancer, but does not report what is the share out of the total number of clients in the HIV clinic. Also, according to the authors a number of HIV-uninfected women were screened for cervical cancer at the clinic, but omitted from evaluation. Therefore, it is difficult to make final conclusions about actual coverage of the initiative.

### Factors affecting service delivery for early detection of cancer

**Fig. 2:**
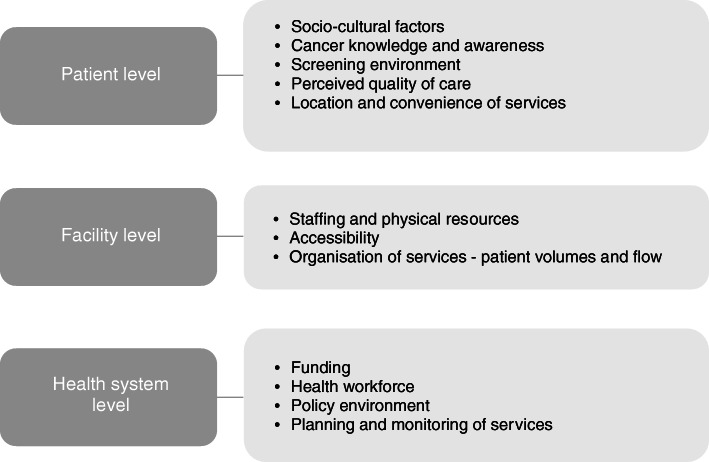
Factors affecting breast and cervical cancer early detection services

We organised the factors affecting the delivery of breast and cervical cancer early detection services into three levels: the patient, the health facility and the health system levels. This analytical framework is illustrated in Fig. 2.

### Patient level factors

At the patient level, the studies in our review identified a range of factors that may influence women’s decision to take part in the cancer screening. These span from practical issues such as lack of time, feeling too ill/tired to participate, needing to tend to family members [18] and indirect costs to access services (e.g. transport to facility, purchasing a health passport [20]), to socio-cultural factors such as needing husband’s approval [18], negative perceptions about preventive care [19], religion and educational barriers [20], embarrassment and modesty [19].

Four articles [18–21] found that raising awareness about both the disease and the screening services had a positive effect on service uptake. Gutnik et al.[18] reported a considerably higher uptake (83%) in women who attended an educational talk prior to being offered the breast cancer screening service compared with those who did not (77%). Additionally, the educational talk led to spill over effects as the newly acquired knowledge was shared by attendees with other members of the community, motivating more women to come for screening [18].

All four of the papers [18–21] reported cancer awareness being considerably low in Malawi. Maseko et al.[21] reported that over half of interviewed women did not know that cervical cancer can be prevented if detected at an early stage, 72% had never heard of the VIA screening test, and 88% did not know any causes of cervical cancer, even though the national cervical cancer screening programme had been running in Malawi for 10 years.

The limited awareness of the disease, screening options and of availability of treatment might be linked to some of the concerns reported by women in the reviewed literature. Kohler et al. [19] reported that 12.5% of interviewed women indicated that before and/or during the breast cancer examination they were afraid of experiencing pain or discomfort, and of receiving an abnormal result. In the study conducted by Gutnik et al. [18] fear of a cancer diagnosis was the reason given for breast cancer screening refusal in 3% of patients. Similarly, Maseko et al. [20] reported that patients were afraid of knowing their health status in the case of cervical cancer screening.

The way screening services were delivered, in terms of physical environment, comfort and professionalism, played an important role in patient’s perceptions of services. Conducting the screening in private rooms [19], which were clean and neat [21], involving female service providers [19], had positive influences on patients, as these elements of the screening environment made them feel more comfortable and not embarrassed to undress. Kohler et al. [19] reported that in general, male providers were also considered acceptable for breast cancer screening and most participants in their study indicated that as long as the provider was well trained they would accept a doctor, nurse or community volunteer. In the case of bundled screening services some respondents preferred doctors or nurses to deliver the test, as the former were perceived to be more knowledgeable and the latter more trustworthy. Patients also appreciated when service providers addressed their questions or concerns and explained what they were doing throughout the exam [19].

Maseko et al. [21] found that when patients had a short distance to reach the health facility from thier home this positively contributed to their satisfaction with the delivery of the screening. Patients who travelled greater than five kilometres to the facility were on average less satisfied with the same service.

Maseko et al. [21] found no significant difference in satisfaction among women screened in different types of facilities. Kohler et al. [19] reported similar results for breast cancer screening. Respondents in their study had no particular preference over type of facilities, whether local health centres, village dispensaries, district hospitals or tertiary hospitals. In general, they believed any health facility serving women (i.e. family planning, maternity, antenatal and paediatric clinics) would be appropriate, further supporting our conclusion that convenience is an important factor influencing the patient’s decision to access screening services in Malawi. However, as reported by Gutnik et al. [18] this was not the case for other types of clinics: breast cancer screening participation rates varied significantly across the five general clinics (*p* = 0.001) in their study, ranging from 71% in the colposcopy clinic to 86% in the sexually transmitted infections clinic.

Furthermore, all women interviewed in the study conducted by Kohler et al. [19] welcomed the idea of a service combining breast and cervical cancer screening, as this would make access to services more efficient and convenient for them, with valuable time and monetary savings.

### Facility level factors

Studies found that lack of personnel was a critical challenge for both breast and cervical cancer control services in Malawi. A review of implementation of the national cervical cancer programme found that 15% of service providers surveyed had not received training, and additionally no staff were permanently allocated to the cervical cancer service [20]. For breast cancer no data were reported on numbers trained by national initiatives or other projects [18]. In addition, lack of resources such as acetic acid and stock-outs of basic medical supplies were substantial impediments, with over 50% of service providers reporting stock-outs [20]. Lack of space was also reported, with most facilities offering screening in busy family planning rooms [20].

Maseko et al. [21] reported that the majority of surveyed health facilities did not conduct the cancer screening on a daily basis, with most only doing so once or twice a week. These restricted opening times acted as a barrier for patients’ access to services. Being given a scheduled appointment beforehand was considered important by patients and was positively related with experienced service satisfaction [21].

In Gutnik et al. 2016 [18] and Pfaff et al. 2018 [22] studies women were recruited for the cancer screening, breast and cervical respectively, at the time of their attendance to the health clinic for other consultations and were offered the screening immediately after these were completed. While this practice was chosen to avoid disrupting mainstream services [18], in both studies it contributed to an accumulation of patients for cancer screening which, coupled with the low staff numbers [18], led to unmanageable patient volumes and long waiting times [18, 22].

The issue of long waiting times was raised also by Maseko et al. 2014 [21], who recorded a minimum waiting time of 3 h for cervical cancer screening in their study, with 46% of women waiting at least 5 h before being attended to. Waiting times had a statistically significant association with service satisfaction.

To address some of these issues and improve patient flows, Pfaff et al. [22] modified the existing Electronic Medical Record (EMR) System used in the HIV clinic where the cervical cancer screening took place to incorporate VIA. While the electronic system helped streamline the movement of patients from the HIV visit room to the VIA room, the authors found little change in the monthly average of women receiving VIA screening for the first time, but the percentage of women who received cryotherapy on the same day increased [22].

### Health system level factors

Inadequate funding has major implications for the delivery of breast and cervical cancer screening services. According to Maseko et al. [20], over half of surveyed facilities said that funding was insufficient. This study also reported a skewed distribution of service providers across Malawi, in favour of urban clinics, even though the majority of the country’s population resides in rural areas. In addition, poor clinical supervision of service providers influenced the effectiveness of service delivery. A retrospective cohort study of cervical cancer screening in Malawi found a high staff turnover in government facilities, with 30% of health workers initially trained under the national cervical screening programme no longer providing cervical cancer screening services at the time of the study [8].

It was also found that, despite the fact that all facilities providing cervical cancer screening were supposed to submit quarterly cervical cancer reports to the Zone Health Support Office (ZHSO), only 4 of the 21 clinics reported doing so^20^. Due to these inadequate reporting systems, it was impossible for the ZHSOs to understand the individual requirements of each clinic, including medical supplies, human resources and infrastructure.

Lastly, there was a lack of awareness and clarity about national policies and guidelines: Maseko et al. found that only 12 out of 41 service providers were aware of the government policy supposed to guide and govern their cervical cancer work [20].

### Referral and treatment services

Mirroring the findings on barriers to screening, the published literature showed that similar barriers affect referral and treatment services. At the patient level, deferring treatment instead of opting for same-day treatment, in the case of precancerous cervical lesions, was a major factor in never returning for treatment [21, 22]. This choice was often driven by socio-cultural issues (e.g. need to discuss cold coagulation treatment with partners first [22]) or financial considerations [21]. Local solutions were implemented in some projects to overcome these challenges, such as phone call reminders and transport reimbursements [18]. At the facility level, disorganised [22] and under-resourced services affected care delivery [8, 20], including shortages of essential equipment such as cryotherapy and Loop Electrosurgical Excision Procedure (LEEP) machines [20]. At the health system level, lack of trained personnel in rural areas affected patient ability to initiate timely referral [20].

## Discussion

The objective of this systematic review was to assess the state of breast and cervical cancer early detection services in Malawi and to explore influencing factors. Researching ways to address these two cancers is of particular importance in Malawi, a country where patients present to health facilities at very advanced stages (particularly for breast cancer), predominantly due to low cancer awareness and lack of accessible screening or control programmes [3, 24]. The picture emerging from the reviewed literature shows that cervical cancer screening and early diagnosis services in Malawi are predominantly delivered under the national cancer control programme, a well-established initiative, yet still suffering from patchy and inefficient implementation. For breast cancer, early detection services are almost non-existent, except for a small number of projects that pilot tested some methods of rolling out these services to communities. The importance of this literature review is that it provides a synthesis of lessons learned so far from these attempts to tackle cervical and breast cancers in Malawi and it offers a systematic overview of factors hindering service delivery, as well as opportunities for improvement.

The first message from this study is that there are various patient-level barriers preventing uptake of screening and early detection services. They range from personal factors such as fear, embarrassment and lack of knowledge/awareness, to issues pertaining to access (long distances) and cultural factors such as negative local beliefs surrounding preventative healthcare. Such factors need to be taken into account by health planners, researchers and implementers aiming to strengthen screening services in Malawi. As demonstrated elsewhere, education plays an important role in overcoming these problems [25, 26], but significant time may be needed to overcome cultural barriers which strongly influence health seeking behaviour in the region [27, 28]. It has been documented that knowledge about cancer differs between rural and urban women [29], so education campaigns may be particularly challenging in a highly rural country such as Malawi. Findings from this literature review show evidence of successful education strategies having positive effects on the rates of women attending screening services. Breast and cervical cancer control programmes in Malawi need to incorporate an educational component in order to maximise uptake, a recommendation which is also echoed in the National Cervical Cancer Control Strategy 2016–2020 [6].

Some strategies have also emerged to overcome prevalent cultural barriers [30, 31]. Embarrassment was successfully prevented by some programmes because screening was conducted in a private environment [18]. Reviewed studies also showed that the screening process can be acceptable and well-received by Malawian women when they can easily and conveniently access the services. Therefore planning for screening programmes should take existing geographical and logistical barriers into consideration, including opening hours of clinics and bundling of screening with other health services. Community engagement and sensitisation are also needed to increase the acceptability and uptake of preventative healthcare, as reported in other countries in the region with similar socio-economic situations [32]. Males, especially heads of households and community leaders, need to be included in any future interventions addressing cultural issues preventing uptake of screening [33].

Secondly, health facilities in Malawi face multiple barriers which prevent them from successfully delivering breast and cervical cancer early detection and screening services. Facility-level shortages of all kinds have been well documented in other areas of the Malawian health sector [13] and reflect its overall poor condition. Our study demonstrated that clinics offering breast and cervical cancer screening and treatment are often understaffed - a common feature of facilities in Malawi [34]. This was partly due to the uneven distribution of the workforce between urban and rural areas, and high staff turnover, with many service providers no longer providing care. The staff who did provide screening services reported lack of motivation and commitment, and inadequate training and supervision. Similar challenges have been documented in other healthcare disciplines in Malawi [35]. Addressing these gaps is essential in view of improving both access and quality of services in Malawi at all levels of health facilities [36], but may be particularly challenging in this resource-limited setting [37].

The final group of barriers to screening relates to weaknesses in the wider healthcare system. These include lack of infrastructure, lack of data to inform planning and inadequate government health expenditure, also documented in other areas of healthcare services in the country [38] and not uncommon in the region [39]. Without data to monitor resources and performance, needs-based planning, and capital and resources from the government, healthcare facilities will be unable to solve their logistical issues that prevent patients receiving an early diagnosis and adequate treatment.

## Conclusion

Breast and cervical cancer together account for over half the cancer burden experienced by women in sub-Saharan Africa, resulting in rapid, high mortality rates. Affordable and appropriate screening and early diagnosis measures exist for low resource settings: visual inspection with acetic acid for cancer of the cervix and clinical breast examination for breast cancer; with thermal-coagulation or cryotherapy for treating precancerous lesions. The reviewed literature reveals the overall poor condition of such services in Malawi. Women in rural areas can rarely access these interventions, because there has not been any coordinated and well-resourced response to the growing burden of these cancers, yet they are the highest mortality-causing cancers in Malawian women. Evidence from the interventions presented in this review can provide guidance for researchers and policy makers on how to improve access to screening and early detection for common cancers. Further interventions should be guided by national cancer response plans, and although some work has been done in the area of cervical cancer [6], implementation, as demonstrated in this literature review, remains challenging. However there is still no policy nor national programme for breast cancer [18] in Malawi.

Finally, whilst Malawi was the focus of this review, the barriers to effective service delivery and mitigation strategies identified in this study may offer useful insights for other countries in the Sub-Saharan Africa region facing similar challenges with regards to resource constraints and patient profiles (largely rural-based and strong socio-cultural influence on health seeking behaviour). This manuscript presents an evidence benchmark for Malawi, future research efforts should have the dual purpose of informing intervention strategies to improve cancer early detection services in Malawi, as well as supporting cross-country analysis to facilitate knowledge sharing and mutual learning among countries with similar challenges.

### Limitations

A limitation of the subject of our review is the low number of rigorous studies identified and their heterogeneity, which made drawing definite conclusions difficult and highlighted the need for more empirical research. However, all identified studies reported similar challenges in the delivery of screening and early diagnosis services, in line with studies regarding other healthcare services in Malawi. Secondly, some articles described different elements of the same intervention, which limits the scale of the evidence base and the generalisability of our conclusions. The quality of retrieved evidence was affected by risk of bias in some cases (see Table 2), ranging from poor data collection systems (raising questions about data quality), to small sample studies in few facilities.

## Data Availability

All data analysed in this review are cited in this published article.

## Appendix

**Table 3:** Detailed search strategy

Embase 12-06-2019		
**Breast cancer screening search**		
Search No.	Query	Results
1	Breast AND neoplasms	575,615
2	Breast tumour	519,737
3	Breast cancer	569,731
4	#1 OR #2 OR #3	622,644
5	‘early cancer diagnosis’	5194
6	Early AND detection AND of AND cancer	61,184
7	‘diagnosis’	5,376,368
8	Diagnostic AND screening AND programmes	1445
9	Diagnostic AND screening	128,611
10	Screening	996,666
11	#5 OR #6 OR #7 OR #8 OR #9 OR #10	6,057,820
12	#4 AND #11	197,498
13	Malawi	10,615
14	Malawian	1433
15	Nyasaland	43
16	#13 OR #14 OR #15	10,745
17	#12 AND #16	31
**Cervical cancer screening search**		
Search No.	Query	Results
1	‘uterine cervix tumor’	26,140
2	Uterine AND cervical AND neoplasms	4174
3	Cervical AND tumour	6052
4	Cervical AND neoplasms	6981
5	Cervical AND cancer	110,347
6	#1 OR #2 OR #3 OR #4 OR #5	130,012
7	‘early cancer diagnosis’	5194
8	Early AND detection AND of AND cancer	61,184
9	‘diagnosis’	5,376,368
10	Diagnostic AND screening AND programmes	1445
11	Diagnostic AND screening	128,611
12	Screening	996,666
13	#7 OR #8 OR #9 OR #10 OR #11 OR #12	6,057,820
14	#6 AND #13	60,592
15	Malawi	10,615
16	Malawian	1433
17	Nyasaland	43
18	#15 OR #16 OR #17	10,745
19	#14 AND #18	53

PubMed 12-06-2019		
**Breast cancer screening search**		
Search No.	Query	Results
1	Breast neoplasms [MeSH Terms]	276,872
2	Breast cancer	385,709
3	(Breast neoplasms [MeSH Terms]) OR (Breast cancer)	385,709
4	Early detection of cancer [MeSH Terms]	21,380
5	Diagnosis [MeSH Terms]	8,178,463
6	Diagnostic screening programmes [MeSH Terms]	23
7	Screening	4,523,284
8	(((Early detection of cancer [MeSH Terms]) OR Diagnosis [MeSH Terms]) OR Diagnostic screening programmes [MeSH Terms]) OR Screening	9,737,844
9	(((Breast neoplasms [MeSH Terms]) OR (Breast cancer)) AND (((Early detection of cancer [MeSH Terms]) OR Diagnosis [MeSH Terms]) OR Diagnostic screening programmes [MeSH Terms]) OR Screening)	194,845
10	Malawi [MeSH Terms]	4772
11	Malawi	8491
12	Nyasaland	8498
13	((Malawi [MeSH Terms]) OR Malawi) OR Nyasaland	8498
14	(((((Breast neoplasms [MeSH Terms]) OR (Breast cancer)) AND ((((Early detection of cancer [MeSH Terms]) OR Diagnosis [MeSH Terms]) OR Diagnostic screening programmes [MeSH Terms]) OR Screening))) AND (((Malawi [MeSH Terms]) OR Malawi) OR Nyasaland)	22
**Cervical cancer screening search**		
Search No.	Query	Results
1	Uterine cervical neoplasms [MeSH Terms]	71,732
2	Cervical cancer	101,035
3	#1 OR #2	101,035
4	Early detection of cancer [MeSH Terms]	21,380
5	Diagnosis [MeSH Terms]	8,178,463
6	Diagnostic screening programmes [MeSH Terms]	23
7	Screening	4,523,284
8	#4 OR #5 OR #6 OR #7	9,737,844
9	#3 AND #8	61,524
10	Malawi [MeSH Terms]	4772
11	Malawi	8491
12	Nyasaland	8498
13	#10 OR #11 OR #12	8498
14	#9 AND #13	37

Ovid MEDLINE 12-06-2019		
**Breast cancer screening search**		
Search No.	Query	Results
1	Breast neoplasms/	270,748
2	Breast cancer.mp.	219,170
3	#1 OR #2	312,335
4	‘Early Detection of Cancer’/	21,297
5	Diagnosis/	17,205
6	Early diagnosis/	24,372
7	Diagnostic screening programmes.mp.	1
8	Mass screening/	97,678
9	Screening.mp.	491,999
10	#4 OR #5 OR #6 OR #7 OR #8 OR #9	535,354
11	#3 AND #10	28,458
12	Malawi/	4766
13	Malawi.mp.	5935
14	Nyasaland.mp.	44
15	#12 OR #13 OR #14	5937
16	#11 AND #15	5
**Cervical cancer screening search**		
Search No.	Query	Results
1	Uterine neoplasms/	71,669
2	cervical cancer.mp.	36,898
3	#1 OR #2	79,060
4	‘Early Detection of Cancer’/	21,297
5	Diagnosis/	17,205
6	Early diagnosis/	24,372
7	Diagnostic screening programmes.mp.	1
8	Mass screening/	97,678
9	Screening.mp.	491,999
10	#4 OR #5 OR #6 OR #7 OR #8 OR #9	535,354
11	#3 AND #10	15,768
12	Malawi/	4766
13	Malawi.mp.	5935
14	Nyasaland.mp.	44
15	#12 OR #13 OR #14	5937
16	#11 AND #15	13

The Cochrane Library 11-06-2019		
**Breast cancer screening search**		
Search No.	Query	Results
1	MeSH descriptor: [Breast Neoplasms] explode all trees	11,655
2	(breast cancer):ti,ab,kw	33,063
3	#1 OR #2	34,039
4	MeSH descriptor: [Early Detection of Cancer] explode all trees	995
5	MeSH descriptor: [Diagnosis] explode all trees	315,302
6	MeSH descriptor: [Diagnostic Screening Programs] explode all trees	0
7	(screening):ti,ab,kw	47,009
8	#4 OR #5 OR #6 OR #7	353,405
9	#3 AND #8	8013
10	MeSH descriptor: [Malawi] explode all trees	319
11	(Malawi):ti,ab,kw	837
12	(Nyasaland):ti,ab,kw	0
13	#10 OR #11 OR #12	837
14	#9 AND #13	0
**Cervical cancer screening search**		
Search No.	Query	Results
1	MeSH descriptor: [Uterine Cervical Neoplasms] explode all trees	1874
2	(cervical cancer):ti,ab,kw	4045
3	#1 OR #2	4667
4	MeSH descriptor: [Early Detection of Cancer] explode all trees	995
5	MeSH descriptor: [Diagnosis] explode all trees	315,302
6	MeSH descriptor: [Diagnostic Screening Programs] explode all trees	0
7	(screening):ti,ab,kw	47,009
8	#4 OR #5 OR #6 OR #7	353,405
9	#3 AND #8	1983
10	MeSH descriptor: [Malawi] explode all trees	319
11	(Malawi):ti,ab,kw	837
12	(Nyasaland):ti,ab,kw	0
13	#10 OR #11 OR #12	837
14	#9 AND #13	3

Global Health 13-06-2019		
**Breast cancer screening search**		
Search No.	Query	Results
1	(breast and neoplasms).sh.	13,617
2	Breast cancer/	25,073
3	Breast cancer.mp.	28,779
4	1 or 2 or 3	29,042
5	Screening.sh.	55,422
6	Diagnosis.sh.	145,385
7	Early diagnosis.sh.	1798
8	Early detection of cancer.mp.	119
9	Diagnostic screening programmes.mp.	0
10	Screening.mp.	116,243
11	5 or 6 or 7 or 8 or 9 or 10	242,276
12	4 or 11	6147
13	Malawi/	5252
14	Malawi.mp.	5470
15	Nyasaland.mp.	5252
16	13 or 14 or 15	5470
17	12 and 16	8
**Cervical cancer screening search**		
Search No.	Query	Results
1	Uterine cervical neoplasms.mp.	206
2	cervical cancer/	13,153
3	Breast cancer.mp.	14,835
4	1 or 2 or 3	14,837
5	Screening.sh.	55,422
6	Diagnosis.sh.	145,385
7	Early diagnosis.sh.	1798
8	Early detection of cancer.mp.	119
9	Diagnostic screening programmes.mp.	0
10	Screening.mp.	116,243
11	5 or 6 or 7 or 8 or 9 or 10	242,276
12	4 or 11	6882
13	Malawi/	5252
14	Malawi.mp.	5470
15	Nyasaland.mp.	5252
16	13 or 14 or 15	5470
17	12 and 16	19

African Journals OnLine (AJOL) 13-06-2019	
Query	Result
Screening AND Malawi	7

## Abbreviations

AIDS: Acquired immunodeficiency syndrome
HIV: Human immunodeficiency virus
CECAP: Cervical cancer control programme
VIA: Visual inspection with acetic acid
SRHR: Sexual and Reproductive Health and Rights Policy
PRISMA: Preferred reporting Items for systematic reviews and meta-analyses
PCC: Population, concept and context
PRESS: Peer review of electronic search strategies MeSH Medical subject headings
AJOL: African journals online
CBE: Clinical breast examination
GRADE: Grading of recommendations, assessment, development and evaluations
EMR: Electronic medical record
ZHSO: Zone health support office
LEEP: Loop electrosurgical excision procedure
